# Integrative pathway-based survival prediction utilizing the interaction between gene expression and DNA methylation in breast cancer

**DOI:** 10.1186/s12920-018-0389-z

**Published:** 2018-09-14

**Authors:** So Yeon Kim, Tae Rim Kim, Hyun-Hwan Jeong, Kyung-Ah Sohn

**Affiliations:** 1 Department of Computer Engineering, Ajou University, Suwon, 16499 South Korea; 2 Department of Molecular and Human Genetics, Baylor College of Medicine, Houston, TX 77030 USA; 3 Jan and Dan Duncan Neurological Research Institute, Texas Children’s Hospital, Houston, TX 77030 USA

**Keywords:** Multi-omics, Integrative analysis, Random walk, Denoising autoencoder, Pathway, Breast cancer, Gene expression, DNA methylation

## Abstract

**Background:**

Integrative analysis on multi-omics data has gained much attention recently. To investigate the interactive effect of gene expression and DNA methylation on cancer, we propose a directed random walk-based approach on an integrated gene-gene graph that is guided by pathway information.

**Methods:**

Our approach first extracts a single pathway profile matrix out of the gene expression and DNA methylation data by performing the random walk over the integrated graph. We then apply a denoising autoencoder to the pathway profile to further identify important pathway features and genes. The extracted features are validated in the survival prediction task for breast cancer patients.

**Results:**

The results show that the proposed method substantially improves the survival prediction performance compared to that of other pathway-based prediction methods, revealing that the combined effect of gene expression and methylation data is well reflected in the integrated gene-gene graph combined with pathway information. Furthermore, we show that our joint analysis on the methylation features and gene expression profile identifies cancer-specific pathways with genes related to breast cancer.

**Conclusions:**

In this study, we proposed a DRW-based method on an integrated gene-gene graph with expression and methylation profiles in order to utilize the interactions between them. The results showed that the constructed integrated gene-gene graph can successfully reflect the combined effect of methylation features on gene expression profiles. We also found that the selected features by DA can effectively extract topologically important pathways and genes specifically related to breast cancer.

## Background

Integrative analysis on multi-omics data to find biomarkers or pathway features highly associated with cancer has received considerable attention [1–6]. Considering the rich information contained in multi-omics data, many studies have investigated the interrelationships among multiple meta-dimensional data for improved biological interpretation and analysis [7–12]. To understand the interaction between different types of genomic features requires more sophisticated modeling and analysis. In particular, the causal relationships between gene expression data and DNA methylation have been extensively studied [13–16]. For joint analysis of gene expression and methylation data in cancer, pathway and subtype information have proven especially useful [17–19]. In this study, we address the problem of pathway-driven integrated analysis of gene expression and methylation data in cancer.

To combine pathway information into genomic analysis and cancer prediction, several methods of inferring pathway activity have been proposed [20–24]. For example, the mean and median of the expression values of pathway member genes can be used for precise cancer classification [24]. In [20], pathway activity inference method of condition-responsive genes (the pathway member genes whose combined expression show optimal discriminative power for the disease phenotype) have been proposed to incorporate pathway information into the precise disease classification. Pathway activity inference approaches using probabilistic inference have been used for combining multiple types of omics data and a better cancer classification [21–23]. However, those existing pathway-based methods simply take pathways as the set of genes and have ignored the topological importance of the hub genes in the pathway network that can be highly associated with diseases. In this respect, Liu, et al. proposed a directed random walk (DRW)-based pathway inference method to identify the topologically important genes and pathways by weighting the genes in the pathway network [25]. Because this original DRW method targeted a single profile of gene expression data, recent approaches have focused on integrating multiple types of data, for example, gene expression and metabolite data [26]. Directed random walk on a gene-metabolite graph (DRW-GM) was performed guided by pathway information, and identified important differential genes and risk pathways in prostate cancer.

**Fig. 1:**
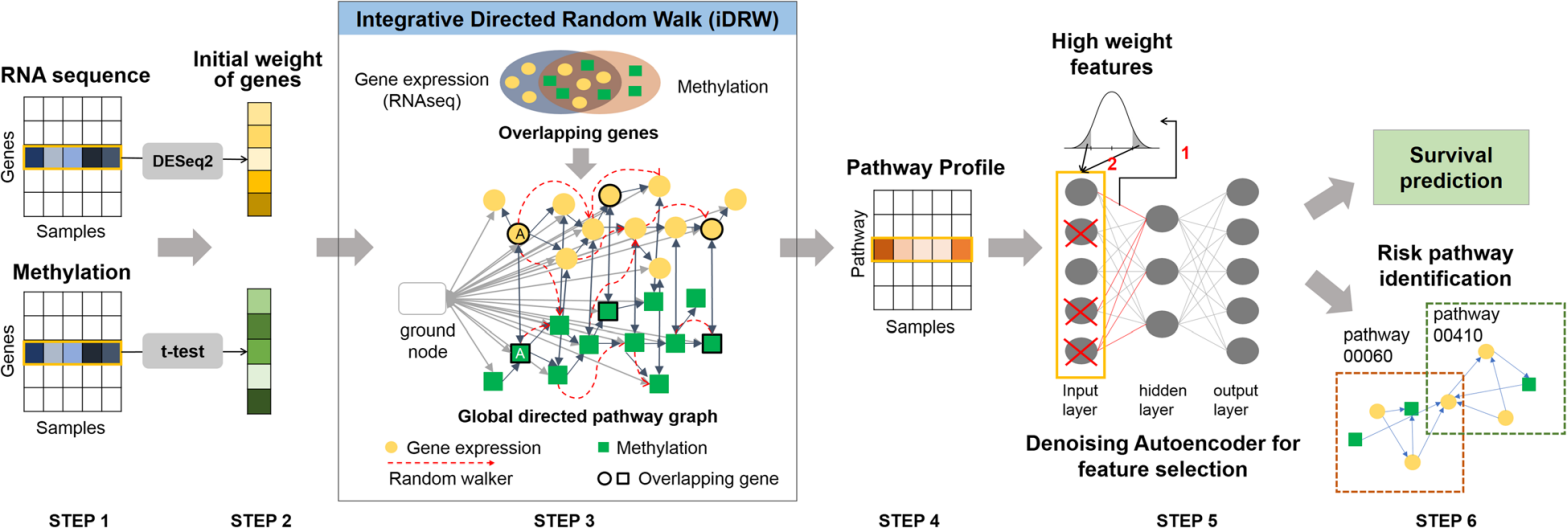
Overview of the proposed integrative pathway-based survival prediction method

In this study, we propose a DRW-based approach on an integrated gene-gene graph especially redefined for gene expression and methylation data in order to extract important pathway and gene features for survival prediction. We first construct an integrated gene-gene graph by adding edges between gene expression and methylation features as well as edges within each profile. In constructing the integrated gene-gene graph, we consider two approaches: one that adds bi-directional edges between expression and methylation features of the same gene that has both profiles, and another that considers only the anti-correlated interactions between the expression and methylation data. For the edges within each single profile, we adopt the pathway-based interaction graph from the previous study [25]. DRW is then performed, which produces the weight values of both expression and methylation features. The initial weights of the gene expression nodes are measured by DESeq2 [27], which is a method for differential gene expression analysis in count data from high-throughput sequencing assays. The methylation feature nodes are initially weighted by using a two-tailed *t*-test between two phenotypes. By using the output from the DRW, a pathway activity profile is computed. In summary, integrative DRW (iDRW) on a graph defined over gene expression and methylation features transforms the combined profile of gene expression and methylation data into a single pathway profile. To further extract important pathway features, we apply a denoising autoencoder (DA) [28] to the pathway profile matrix. DA has proven to be effective in selecting robust features against input noise and extracting more specific cancer-related pathways or genes [29–31]. The resulting features are validated on a survival prediction task of breast cancer patients. The topologically significant pathways and pathway member genes are identified and analyzed as well. The overall process of the proposed approach is illustrated in Fig. 1.

The pathway features selected with our scheme are based on gene expression and methylation features as well as interactions between the two. These extracted pathway features are effective at improving the prediction performance when compared to the gene-based profile or other pathway-driven methods. We also reveal that the iDRW method with a denoising autoencoder selects a more cancer-specific pathways or genes as compared to that directly selected by the iDRW method.

## Methods

### Dataset

Gene expression and DNA methylation data of 868 breast cancer patients were obtained from the TCGA dataset of the Broad Institute GDAC Firehose [32]. Gene expression data from RNA sequencing consisted of 17,673 genes, which are upper-quartile normalized RSEM count estimates in the Broad Institute GDAC Firehose [33]. DNA methylation data were obtained as a gene-level feature of 17,037 genes by selecting the probe having a minimum correlation with expression data for each gene [34]. We removed genes in which more than half had gene expression values of 0. In contrast to gene expression data, 5134 missing values were present in the methylation data. To impute missing values, we replaced them with a median of the corresponding patient’s data. For each breast cancer patient, the vital status and survival days were recorded. Among 868 patients, we extracted 568 samples that had both RNA sequencing and methylation data. We removed patients whose survival days were not recorded or wrongly so as negative values. In this study, we split the patients into good (> 3 years) and poor (≤ 3 years) groups with respect to their survival days [35]. Patients who were living (vital status reported as 1) but whose survival days were less than 3 years were removed. In total, 465 samples were divided into two groups of 218 good and 247 poor. Finally, the gene expression and methylation data were normalized for the mean to be 0 and standard deviation to be 1 over all samples.

### Pathway-based global directed integrated gene-gene graph

To transform each gene profile into a pathway profile, a DRW-based method was performed on a global directed gene-gene graph, which was constructed based on both 150 metabolic and 150 non-metabolic KEGG pathways [25]. Interactions between genes in the global directed graph were manually drawn from the KEGG database [36] by researchers in [25]. The global directed graph contained 4113 genes and 40,875 directed edges. Details regarding the construction method of the global directed graph are provided in [25].

To define the directed graph across gene expression and methylation data, we first included all edges in the global directed graph from [25] within each profile. In addition, the interactions between 16,454 overlapping genes in the two profiles were defined in the global directed graph. As most of the methylation profiles inhibited the genes in the gene expression data [37], we experimented with two cases. First, we assigned bi-directional edges to all overlapping genes between gene expression and methylation data. Second, we only assigned the edge when the expression and methylation values of the same gene were anti-correlated. Correlation was measured by the Pearson correlation and significance test of a correlation coefficient was performed. The correlation coefficient with a negative value and *p*-value of a significant test < 0.05 meant that the methylation profile might inhibit the corresponding gene expression. The final integrated gene-gene graph contained 4113 genes as nodes, which were either from the gene expression data or methylation profiles. The number of directed edges in the graph was 88,440 when all overlapping edges were added and 81,750 (the removal of edges is about 7.6% of all overlapping edges) when only the anti-correlated edges were added.

### DRW-based method on an integrated gene-gene graph

We utilized the recently proposed DRW method (DRW-GM) [26] to integrate information in a graph constructed from multiple profiles. To perform random walk, the initial weights of the genes should be assigned. As the DRW-GM method is specifically designed to integrate gene expression profiles and metabolomic profiles, the weights of the genes were modified for the graph from the gene expression and methylation profiles. For each gene profile, *W*_0_ is constructed as:

$$ {W}_0=-\mathit{\log}\left({w}_g+\epsilon \right) $$

where *w*_*g*_ is the weight of the gene *g* in the directed integrated gene-gene graph *G*, and *ϵ* = 2.2*e*^−16^*.* The weight of the gene is the *p*-value from either a two-tailed *t*-test for the methylation profiles or a DESeq2, which is a method for differential gene expression analysis based on negative binomial distribution for RNA sequence genes [27]. Each gene weight vector is normalized to scale the range between 0 and 1. Finally, *W*_0_ is *L*_1_-normalized to a unit vector. A random walker starts on a source node *s* and transits to a randomly selected neighbor or returns to the source node *s* with a restart probability *r* at each time step *t*. The DRW method is formally defined as:

$$ {W}_{t+1}=\left(1-r\right){M}^T{W}_t+r{W}_0 $$

where *W*_*t*_ is the weight vector in which the *i*-th element represents the probability of being at node *i* at time step *t*; *M* is a row-normalized adjacency matrix of the directed integrated gene-gene graph *G*; *r* is the restart probability, which is set to 0.7 (as it was previously shown that the performance of the DRW method is not sensitive to the varying *r* [25]), and *W*_0_ is the initial weight vector of genes in the graph *G*. At each time step, *W*_*t*_ is updated and guaranteed to converge to a steady state *W*_∞_ [38] when∣*W*_*t* + 1_ − *W*_*t*_∣< 10^−10^*.*

### Pathway activity inference

For a *j*-th pathway *P*_*j*_ containing *n*_*j*_ differential genes $\left({g}_1,{g}_2,\dots, {g}_{n_j}\right)$ whose *p*-value (*w*_*g*_) is < 0.05, the pathway activity is defined as:

$$ a\left({P}_j\right)=\frac{\sum_{i=1}^{n_j}{W}_{\infty}\left({g}_i\right)\ast score\left({g}_i\right)\ast z\left({g}_i\right)}{\sqrt{\sum_{i=1}^{n_j}{\left({W}_{\infty}\left({g}_i\right)\right)}^2}} $$

where *W*_∞_(*g*_*i*_) is the weight of gene *g*_*i*_ from the DRW method, *z*(*g*_*i*_) is the normalized expression vector of *g*_*i*_ across overall samples, and *score*(*g*_*i*_) is either a *log*_2_ *fold change* from the DESeq2 [27] analysis if *g*_*i*_ is a gene from the gene expression data, or a *sign*(*tscore*(*g*_*i*_)) from two-tailed *t*-test statistics if *g*_*i*_ is a gene with the methylation feature. For DESeq2 in the gene expression data, *log*_2_ *fold change* indicates the extent to which gene expression values have changed between groups of samples. For each pathway, the pathway activity is computed from the normalized gene expression values for each sample, which corresponds to a pathway profile. As a result, the pathway profile is used as an input to a classification model.

### Feature selection and ranking strategy

To select pathway features, the pathways are first scored by the weight matrix from DA [28]. Given an input *x* ∈ ℝ^*d*^ that is a feature vector and corrupted input $\overset{\sim }{x}\in {\mathrm{\mathbb{R}}}^d$ that is perturbed by a random binomial error, $\overset{\sim }{x}$ is mapped to a hidden representation *y* ∈ ℝ^*p*^ as follows:

$$ y=s\left(W\overset{\sim }{x}+b\right) $$

where *s* is a sigmoid activation function, *W* is a weight matrix that is randomly initialized depending on its input and hidden layer size, *b* is a bias, and *y* is a latent representation of the encoded $\overset{\sim }{x}$ by the encoder. *y* is then used as an input into a decoder to reconstruct *z* as follows:

$$ z=s\left({W}^Ty+{b}^T\right) $$

Here, *z* C input of *x* given *y*. To calculate the reconstruction error, we used a mean squared error, not the cross-entropy as the scale of our data was not in [0, 1]. *L*(*xz*)*,* which is the loss on the reconstruction of the original input *x* from *z*, is defined as:

$$ L(xz)=\frac{\parallel x-z{\parallel}^2}{2} $$

For feature importance scoring purposes, we used a single hidden layer because the input features are scored by the weight matrix between input and hidden layers, and the more abstract features are selected when using the more number of hidden layers which can lead to lose pathway information. Note that the purpose of using DA in this study was primarily for feature selection than for accurate reconstruction of the original input. To rank the pathway features, we first trained the DA to obtain the weight matrix between input and hidden layers. The weight of each input feature was then defined as the mean value of the weight vector of the input node to all hidden nodes. We experimented with a varying number of hidden nodes (50, 100, 150, 200). As the number of hidden nodes did not greatly affect the list of selected pathway features and the final classification performance, the number of hidden nodes was set to 200. In the experiments, the selected pathway features from DA combined with the iDRW method (iDRW+DA) were compared with those obtained using the iDRW method. The pathway features were ranked by their *p*-values from the *t*-test of pathway activities across samples with the iDRW method. Therefore, the ranked features by the iDRW+DA method were selected to best fit the classification model using a greedy search as performed in [25].

### Classification performance evaluation

We performed a logistic regression analysis using the extracted features. A 5-fold cross validation was conducted to evaluate the classification performance. We first divided the entire samples into five folds. We then trained the regression model using four folds and validated the performance using the remaining fold. For each fold, the top-*N* pathway features that yielded the best classification performance were selected; this was measured by area under the curve (AUC) and the accuracy. AUC is the area under the Receiving operating characteristic (ROC) curve evaluating the trade-off between true positive rate (sensitivity) and false positive rate (1- specificity) and the accuracy measures the proportion of true positives and true negatives; the more AUC and the accuracy is, the better the trained regression model classifies the test samples into good and poor group. To select the best pathway features, we repeated the entire cross validation process 10 times and assessed the pathway features that appeared more than three times in a union of 50 feature sets. Finally, the average AUC and accuracy after 10 repeats of the process using five folds was used as a final classification performance.

## Results

### Performance comparison on a single type of feature data

**Fig. 2:**
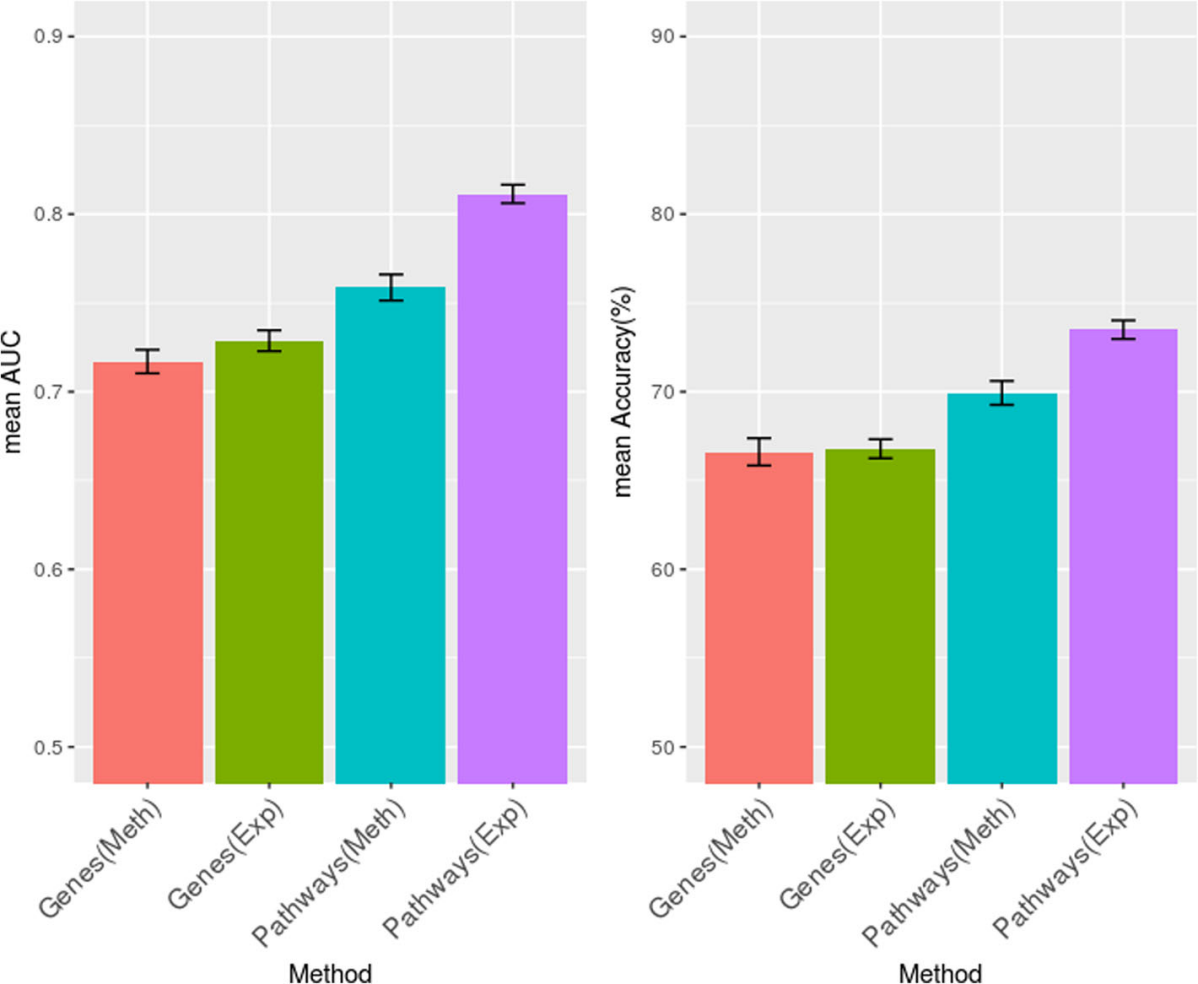
Classification performance comparison between a single type of feature data and converted pathway profiles by DRW in terms of mean AUC (left) and mean accuracy (right) after 10 repeats of 5-fold cross validation process. Gene and pathway profiles with gene expression and methylation profiles were evaluated. The pathway profiles were obtained by the original DRW method. Error bars represent the standard error of the mean values

To check the utility of the pathway profiles obtained using the DRW method, we first experimented with each single-layered feature data. The performances were evaluated using four types of data: RNA-seq gene expression profile, methylation profile, RNA-seq pathway profile, and methylation pathway profile. The pathway profiles were obtained by the original DRW method. The classification performance was evaluated using the selected top-*N* pathway features ranked by their t-test scores. For a fair comparison, top-*N* genes of the gene profiles were also ranked by their DESeq2 or t-test scores. Note that the genes and pathways are weighted via two-group (good and poor groups) comparison that is considered as a supervised learning task. Figure 2 shows the average AUC and the accuracy from a 5-fold cross validation measured using a logistic regression model. As shown in Fig. 2, the overall performance using the pathway profiles from the DRW method was better than that when using the gene profiles. These findings reveal that the pathway features extracted using the DRW method can improve the prediction performance when compared to the gene features. We also determined that the performance difference between RNA-seq data and the methylation profile was considerable when using pathway profiles. This means that gene expression plays a more critical role in survival prediction in a breast cancer patient group than does a methylation profile. Moreover, this difference was particularly remarkable when raw feature values were transformed into pathway features.

### Performance comparison of the pathway-based prediction methods on combined feature data

**Fig. 3:**
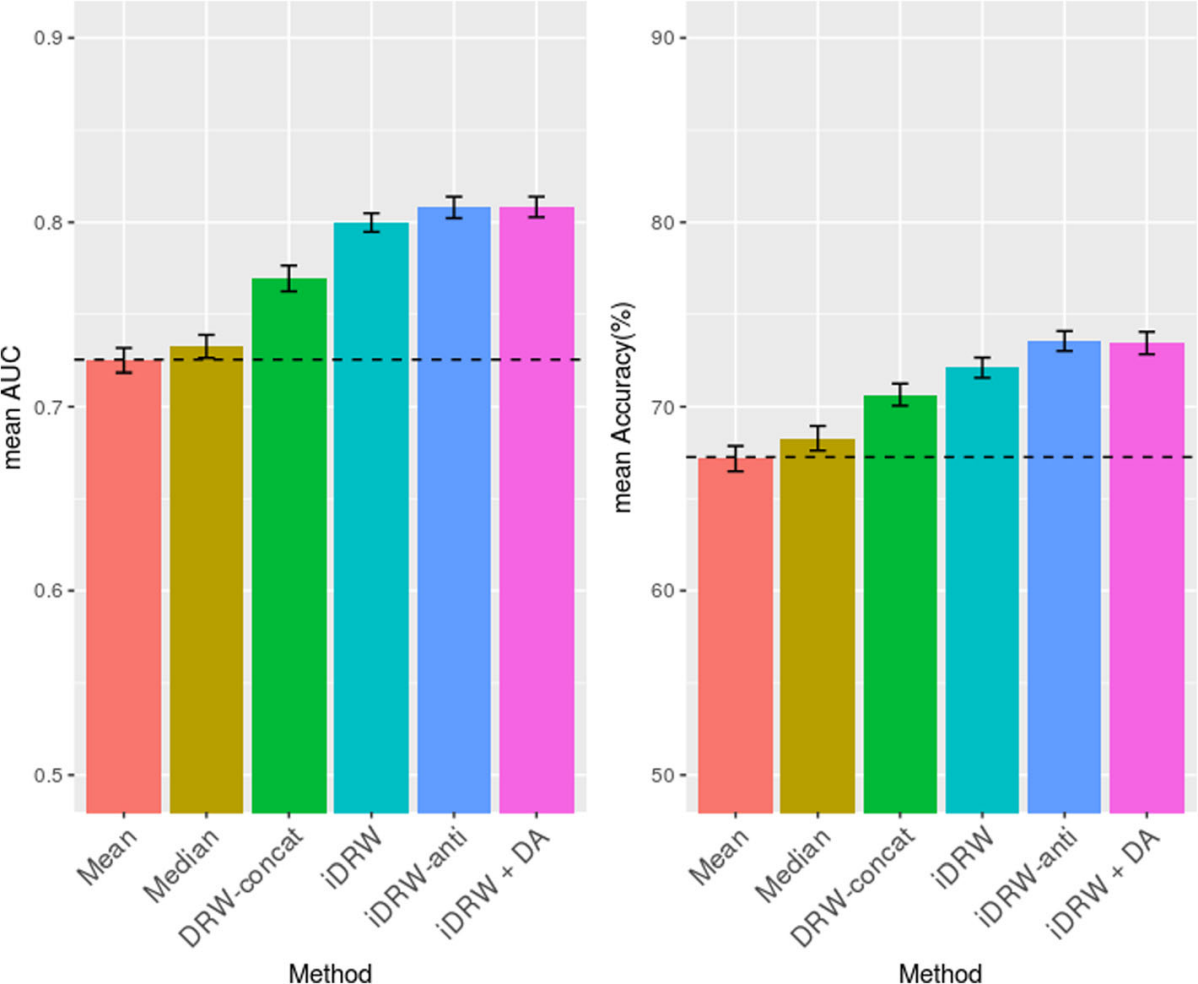
Classification performance comparison of the pathway-based prediction methods on the combined feature data. Mean AUC (left) and mean accuracy (right) after 10 repeats of 5-fold cross validation process are shown. Error bars represent the standard error of the mean values

To show the utility of the proposed method on the combined feature data, we compared different pathway-based prediction methods on the combined RNA-seq and DNA methylation data (Fig. 3). First, we simply employed means (Mean) and medians (Median) of the expression values of the significant pathway member genes to construct a pathway profile matrix. To show the utility of the integrated gene-gene graph, we also assessed the performance when the pathway profiles obtained from the RNA-seq and methylation data were concatenated (DRW-concat). In this method, we used the DRW method to obtain pathway profiles but the interaction of the RNA sequence and methylation data were not considered. The last three results shown in Fig. 3 are from the pathway profiles obtained by the proposed DRW method on the integrated gene-gene graph. As a baseline, the classification performance over the concatenated RNA-seq and methylation profile without using pathway information is shown as a dotted horizontal line in Fig. 3. All performances of the iDRW-based methods outperformed the simple concatenation of the DRW method and the baselines, as expected.

These results reveal that the interactions between gene expression and methylation profiles have considerable joint effect on the integrated gene-gene graph and survival prediction. Regarding the construction of the integrated graph, we first linked all the nodes of the same gene between RNA-seq and methylation profiles (iDRW). Second, we only considered the anti-correlated interactions (iDRW-anti). The classification performance of iDRW combined with the DA (iDRW+DA) was the best, whereas the performance difference between the three iDRW methods was marginal.

### Identification of significant pathways and genes in breast cancer

**Fig. 4:**
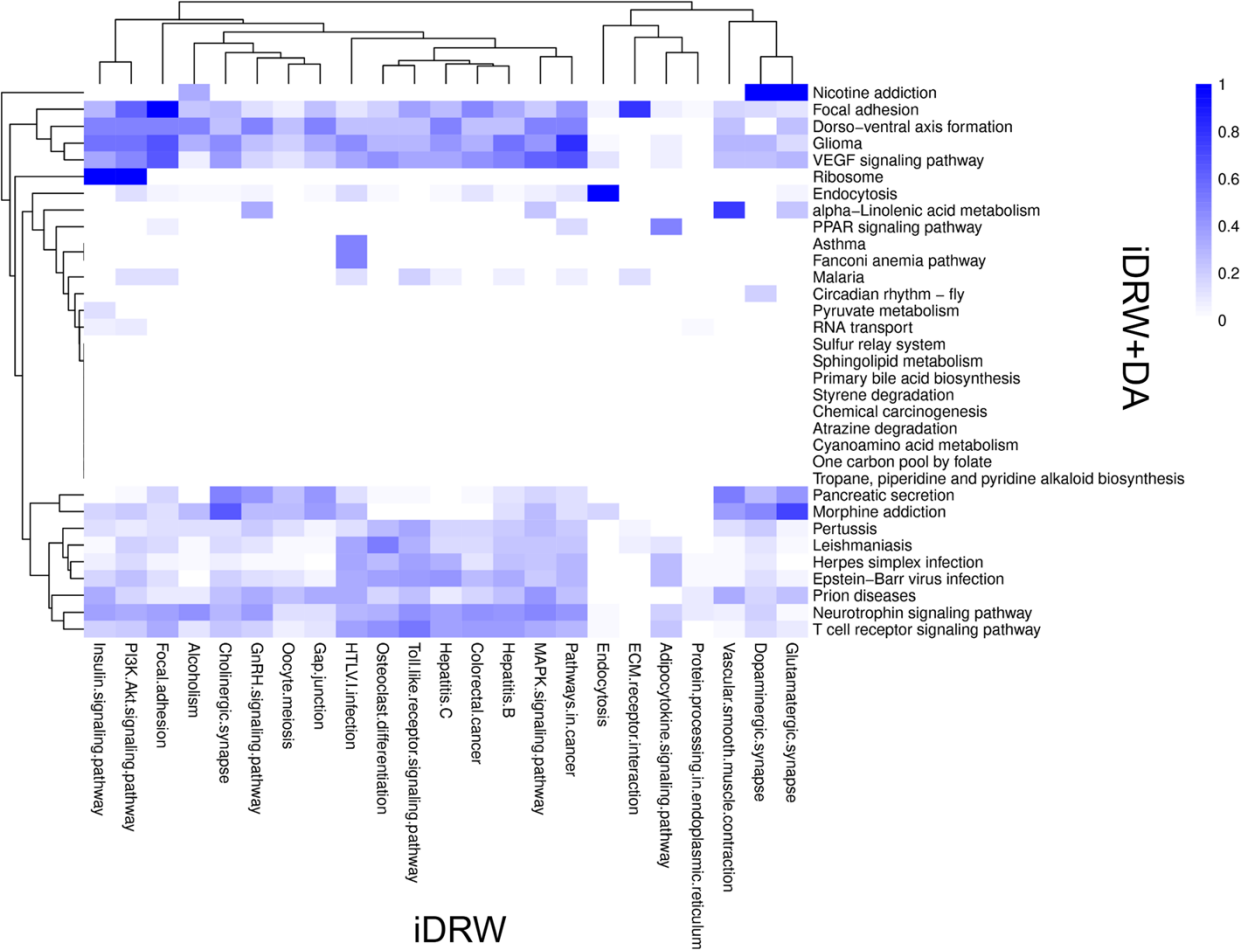
Heat-map for comparing selected pathways by the iDRW and iDRW + DA methods. Each cell represents similarity using Simpson coefficient between two lists of differentially expressed genes and methylated genes from a pair of pathways selected by each method. Note that the rows and columns represent selected pathways by iDRW+DA and the iDRW method, respectively, and are clustered via hierarchical clustering with complete-linkage method

**Fig. 5:**
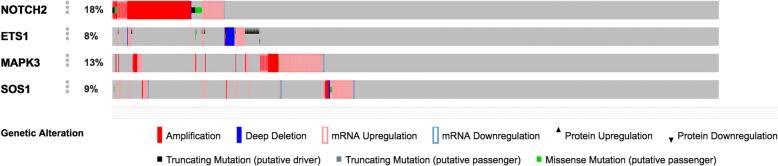
Genetic alterations for the four genes in the dorso-ventral axis formation pathway from the Breast Invasive Carcinoma dataset in cBioPortal (http://www.cbioportal.org/)

In our study, we could extract significant pathway features from both the iDRW outputs and the iDRW+ DA. Figure 4 compares the lists of selected pathways from both the iDRW and the iDRW+DA as a heatmap. Each cell in the heatmap represents similarity using the Simpson coefficient [39] between two lists of differentially expressed genes and methylation sites from a pair of pathways. It measures how many genes were overlapped between the selected pathways by the iDRW and the iDRW+DA. The rows and columns in the heatmap represent selected pathways by DA and the iDRW method, respectively. Note that the iDRW method weighted the pathway features by the two-tailed t-test statistics, whereas the iDRW+DA used the weight matrix between the input nodes and hidden nodes in DA. We observed that the pathways selected by the iDRW method had similar patterns to those from iDRW+DA, which are marked as colored rows in the heatmap. This means that the iDRW method can detect general and non-specific pathways such as MAPK signaling pathway (86 genes), pathways in cancer (86 genes), and endocytosis (47 genes). However, iDRW+DA identified dorso-ventral axis formation as a top-scoring pathway which is an extremely specific pathway and contains four differentially expressed genes: ETS proto-oncogene 1, transcription factor (ETS1); notch 2 (NOTCH); mitogen-activated protein kinase 3 (MAPK3); and SOS Ras/Rac guanine nucleotide exchange factor 1 (SOS1). The dorso-ventral axis formation is related to the Wnt signaling pathway [40]. Wnt signaling pathway is one of the closely associated pathways with cancer [41]. We also found that approximately 40% of patients (439 of 1098) showed genetic alterations for the four genes in the pathway from the Breast Invasive Carcinoma dataset in the cBioPortal (http://www.cbioportal.org/), as shown in Fig. 5. Moreover, the DisGeNET database (http://www.disgenet.org), which shows relations between genes and diseases, indicates that those genes are associated with cancer-related diseases or disorders such as precancerous conditions (umls: C0032927), follicular thyroid carcinoma (umls: C0206682), and tumor initiation (umls: C0598935). We did not identify any strong evidence of association with pancreatic secretion (KEGG ID: hsa04972). However, we found that 13 genes in the pancreatic secretion pathway may regulate blood circulation as a means of releasing nucleic acids [42]. The circulating nucleic acids by the biological process can be a biomarker of breast cancer. Based on our findings, we can hypothesize that the top-ranked pathways can be directly associated with the survivability of breast cancer patients given additional biological experiments.

**Fig. 6:**
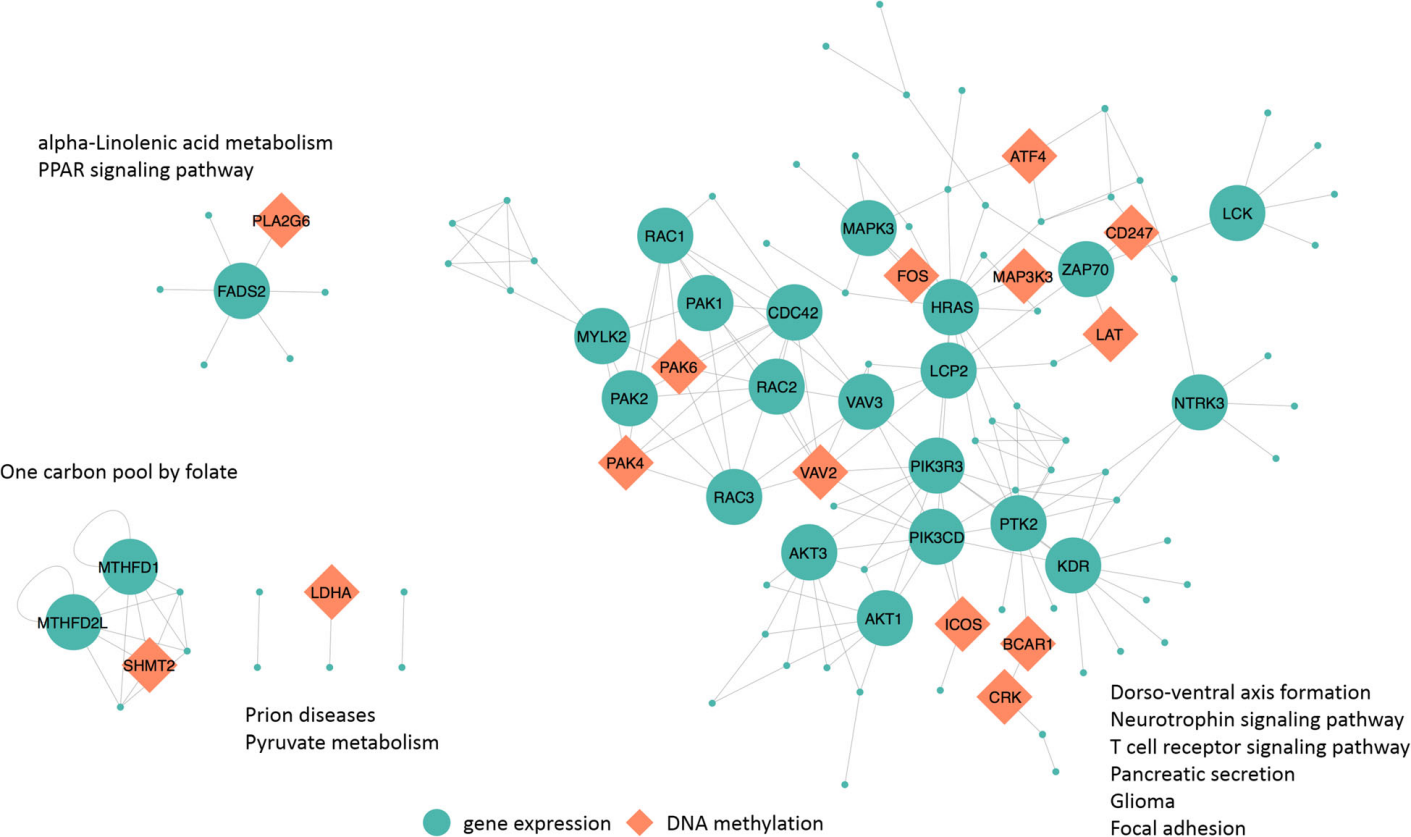
Pathway-based gene-gene interaction network between gene expression profiles and DNA methylation features extracted by iDRW + DA. The genes in the top-10 pathways are shown; the hub genes whose degree is greater than 4 in the gene expression data (green) and genes that are detected in differential methylation regions (orange) are emphasized in different colors

One of the advantages of our method is that it can obtain both differentially expressed genes from gene expression data as well as differentially methylated genes in each pathway. Thus, we can perform a joint analysis of the gene expression and methylation data. Table 1 shows the risk-active pathways selected by the proposed iDRW+DA method. The pathways that appear more than five times during 50 iterations are shown, and the number of significant pathway member genes from the gene expression and methylation data are also reported. The top-ranked pathways (i.e., dorso-ventral axis formation, pancreatic secretion, and neurotrophin signaling pathway) are reported as breast-cancer-related pathways as shown above. The genes in the top-10 pathways in Table 1 are also visualized in the gene-gene network shown in Fig. 6. The hub genes in the network play a crucial role in pathways selected by both the iDRW+DA method and the iDRW method. For example, MAPK3, transforming protein p21 (HRAS), and v-akt murine thymoma viral oncogene homolog 1 (AKT1) were all reported as highly related to the MAPK signaling pathway (KEGG ID: map 04010) known to be associated broadly with many cancers [43, 44]. In addition, PTK2 protein tyrosine kinase 2 (PTK2), phosphatidylinositol 3-kinase regulatory subunit gamma (PIK3R3), and phosphatidylinositol-4,5-bisphosphate 3-kinase catalytic subunit delta (PIK3CD) are shown to be related to pathways in cancer (KEGG ID: map 05200) [9]. Additionally, we investigated the association between the genes in the network and breast cancer using a gene-disease association (GDA) score from DisGeNET database. Note that the hub genes whose degrees in the network are greater than 4 and those genes detected in differential methylation regions are selected (which are colored in Fig. 6). Based on these criteria, 38 genes are used as input to the DisGeNET database. The GDA score above 0.2 for a gene can be interpreted to mean that it is strongly related to the disease, and the GDA score of a gene above 0 reveals that an association between that gene and the disease may be found in public databases and publications. Moreover, if the GDA score for a gene is 0, then no reports exist in any database or literature showing evidence of association between the gene and the disease. According to the GDA scores, 73.69% of hub genes (28 of 38) have GDA scores above 0 for breast cancer-related diseases, and we can claim that among hub-genes in the network, these genes are highly related to the breast cancer-related diseases. Table 2 summarizes the top-5 genes (as ranked by GDA scores from the DisGeNET database) that are associated with each disease. Based on these results, we can conclude that the genes and pathways detected by the proposed iDRW+DA method are related to breast cancer.

**Table 1:** Risk-active pathways identified by the proposed method (iDRW+DA)

Pathway ID	Pathway name	Frequency^a^	Total genes^b^	DE genes	DM genes
map 04320	Dorso-ventral axis formation	10/50	27	4	0
map 04972	Pancreatic secretion	8/50	65	26	3
map 04722	Neurotrophin signaling pathway	7/50	90	47	3
map 05020	Prion diseases	7/50	30	12	0
map 00670	One carbon pool by folate	5/50	33	6	1
map 00592	alpha-Linolenic acid metabolism	5/50	23	8	1
map 00620	Pyruvate metabolism	5/50	96	7	1
map 03320	PPAR signaling pathway	5/50	61	13	1
map 04660	T cell receptor signaling pathway	5/50	85	52	8
map 04510	Focal adhesion	5/50	148	83	11
map 03010	Ribosome	5/50	143	1	0
map 05214	Glioma	5/50	52	27	0
map 04711	Circadian rhythm - fly	5/50	8	4	1
map 00960	Tropane, piperidine, and pyridine alkaloid biosynthesis	5/50	26	1	0

^a^Frequency: the number of times the pathway has been selected over 10 times of 5-fold cross validation process (50 iterations)

^b^Total genes: the number of genes mapped to the pathway in the KEGG database

Note that the number of differentially expressed genes (DE genes) and differentially methylated genes (DM genes) are also shown (*p*-value of DESeq2 or *t*-test < 0.05)

**Table 2:** Top-5 genes ranked by GDA scores from the DisGeNET database (http://www.disgenet.org/) that are associated with breast-cancer-related diseases

Disease ID	Disease	Gene	GDA score
C0678222	Breast Carcinoma	AKT1	0.2418
		PIK3CD	0.0448
		MAPK3	0.0118
		HRAS	0.0077
		BCAR1	0.0074
C0006142	Malignant neoplasm of breast	AKT1	0.2420
		PIK3CD	0.0475
		KDR	0.0119
		MAPK3	0.0110
		PAK1	0.0095
C3539878	Triple Negative Breast Neoplasms	PIK3CD	0.0047
		AKT1	0.0022
		AKT3	0.0011
		MAPK3	0.0011
		KDR	0.0008

## Discussion

The selected pathways by iDRW+DA showed different patterns in comparison with the iDRW method. As the heatmap in Fig. 4 shows, only two pathways of Focal adhesion (KEGG ID: map 04510) and Endocytosis (KEGG ID: map 04144) were identified by both the iDRW+DA method and the iDRW method. In the iDRW+DA method, the genes in the pathways of sphingolipid metabolism (KEGG ID: map 00600), one carbon pool by folate (KEGG ID: map 00670), and chemical carcinogenesis (KEGG ID: map 05204) were detected and previous studies reported that these pathways are associated with breast cancer. The pathway of sphingolipid metabolism is activated by the steroid hormone estrogen. Estrogen includes a variety of cytoplasmic second messengers linked to a multitude of tissue-specific effects, and Sukocheva et al. reported that this hormone triggers the sphingolipid signaling cascade in various tissues, including breast cancer [45]. We also identified chemical carcinogenesis (KEGG ID: map 05204) using our method. In many cases, chemical and physical agents play a critical role in cancer induction, and one study shows that diethylstilbestrol (DES) and bisphenol A (BPA) are estrogen-like endocrine disruption chemicals that induce continual epigenetic changes affecting emerging breast cancer [46]. Moreover, many studies revealed that one carbon pool by folate (KEGG ID: map 00670) is related to cancer. Experiments revealed that one carbon pool by folate is upregulated in a cancer cell line [47]. Furthermore, Shuvalov et al. reported cancer-related metabolism is a hallmark of cancesr. In particular, one-carbon metabolism is reported as the keystone of them all [48]. Thus, we can conclude that the proposed iDRW+DA method contributes to identifying more specific cancer-related pathways, whereas the iDRW method tends to find generally important pathways for cancers. The main difference between the iDRW and iDRW+DA methods is the pathway features ranking strategy. Taken pathway profiles as an input, the pathways are ranked by the t-test statistics (iDRW) or the weight matrix of DA (iDRW+DA). Denoising process of DA can differentiate the features more and discover interesting structure in the input [28]. As it is shown that DA is effective at capturing more distinctive features by learning latent representations of the input [28], we can observe that the iDRW+DA method detects more cancer-specific pathways despite that the performance difference between iDRW and iDRW+DA methods was marginal.

## Conclusions

In this study, we proposed a DRW-based method on an integrated gene-gene graph with expression and methylation profiles in order to utilize the interactions between them. DA-based feature selection was also employed to discover more cancer-specific genes and pathways. The results showed that the constructed integrated gene-gene graph can successfully reflect the combined effect of methylation features on gene expression profiles. The classification performance of the methods showed that pathway-based prediction outperforms gene-based methods. We also found that the selected features by DA can effectively extract topologically important pathways and genes specifically related to breast cancer. Although the classification performance improvement by DA was found to be marginal in our study, DA can extract specific cancer-related biomarkers and facilitate the analysis of biologically meaningful features. The proposed method also identified known breast-cancer-related genes and risk-active pathways successfully. As the integrated gene-gene graph utilized the pathway information using multi-omics data, our study showed that an effective joint analysis on gene expression and methylation data is possible under our framework.

## Abbreviations

AUC: Area under the curve
DA: Denoising autoencoder
DRW: Directed random walk
GDA: Gene-disease association
